# Associations of serum cystatin C with depressive symptoms and suicidal ideation in major depressive disorder

**DOI:** 10.1186/s12888-021-03509-3

**Published:** 2021-11-17

**Authors:** Ting Sun, Qian Chen, Yan Li

**Affiliations:** Department of Clinical Laboratory, Renmin Hospital of Wuhan University, No. 99 Zhangzhidong Road, Wuchang District, Wuhan, 430060 China

**Keywords:** Depression, Serum, Cystatin C, Depressive symptoms, Suicidal ideation

## Abstract

**Background:**

Individuals with major depressive disorder (MDD) have high suicidal ideation. There is evidence that serum cystatin C (Cys C) may be involved in the pathophysiology of MDD. The present study aimed to investigate Cys C concentration in patients with MDD and clarify its possible association with depressive symptoms and suicidal ideation.

**Methods:**

An online cross-sectional survey of 159 patients diagnosed with MDD was conducted. Serum Cys C levels were measured using ADVIA 2400 biochemical analyzer. The 24-item Hamilton Depression Scale (HAMD-24) was administered to evaluate the depressive symptoms. Generalized linear regression, logistic regression and restricted cubic spline models were used to examine the association of serum Cys C levels with depressive symptoms and suicidal ideation.

**Results:**

Serum Cys C levels were higher in MDD patients than in controls (*p* = 0.001) and were positively associated with scores on HAMD-24 in unadjusted (gender distribution, age, smoking, alcohol consumption, family history of depression and traumatic life events; (*p* = 0.003) and fully adjusted linear regression model (*p* = 0.005). The fully adjusted regression coefficient with 95% confidence intervals for serum Cys C levels and HAMD-24 score was 30.339 (9.602 to 51.077). The level of Cys C in the suicidal ideation (SI) group was significantly higher than that in the non-suicide ideation (non-SI) group (*p* = 0.001). Serum Cys C levels were positively associated with suicidal ideation in each logistic regression model (all *p* <  0.05).

**Conclusion:**

Serum Cys C levels were elevated in MDD patients and appeared to be positively correlated with depressive symptoms and suicidal ideation. These findings suggest that the dysfunction of Cys C may be involved in the severity of depression and in the pathophysiological process of MDD. Thus, regulation of serum Cys C could potentially be an effective predictor of the severity of depression and potentially, play a role in reducing the risk of suicide in MDD patients.

## Introduction

Major depressive disorder (MDD), with profound impairment in cognitive and social functioning, is a common and complex psychiatric disorder characterized by high morbidity [1, 2], high disability rate [3], and high suicide rate [4, 5]. According to the World Health Organization (WHO), the global prevalence rate of depression is more than 4%; with the number of people suffering from depression exceeding 350 million at present. A substantial portion of MDD patients, approximately 4 to 10.6% may eventually die by committing suicide, which not only brings great pain to patients and their families, but also imposes huge economic burden on society [6]. Therefore, wide-ranging efforts have been made to explore the biological mechanisms of depression [7, 8].

Although the etiology and pathogenesis of depression have not been fully understood, consistent and robust empirical evidence suggests that neuronal injury [9] and immune inflammation [10–14] are important factors associated with depression. As a major cysteine protease inhibitor in the brain, Cys C is closely related to neuronal damage [15] and immune inflammation [16]. Therefore, recently, increasing interest in recent years has focused on the relationships between serum Cys C and central nervous system diseases, especially depression.

Cys C, a cysteine protease inhibitor contained in all nucleated cells, is encoded by the CST3 gene, secreted in blood, cerebrospinal fluid and extracellular space [17]. Cys C in the brain is mainly derived from astrocytes [15, 18, 19], and the reduced number or dysfunction of astrocytes may cause or somehow contribute to MDD symptomatology [20, 21]. In addition, reduced glial cell volume in the limbic system and other cortical regions may also lead to changes in neuron size and density in mood disorders, which may be closely related to the development of depression [22].

Multiple lines of evidence highlight that Cys C plays an important role in neuronal injury and dysfunction, and is closely related to cognitive dysfunction [23]. Its possible mechanisms include: (1) High Cys C can reduce the activity of cathepsin and cause vascular endothelial cell damage through inflammatory reaction [24]. (2) Cys C and β-amyloid may co-deposit in the micro vascular wall of the brain, which may increase cerebral vascular injury and thus aggravate the occurrence and progression of cognitive dysfunction [25]. (3) Moreover, Cys C is also engaged in apoptosis, oxidative stress, neuronal regeneration and neurodegeneration, which may impair cognitive function [26]. Numerous studies have reported a relationship between serum Cys C and neurodegenerative diseases such as Alzheimer’s and Parkinson’s disease [27, 28], suggesting that high serum levels of Cys C can accelerate the development of these diseases. In addition, similarly to what has been previously reported, patients with higher Cys C concentration appear to present with high risk of depression [29, 30]. However, in previous studies the majority of the participants were elderly people, with few studies exploring changes in Cys C levels in depressed patients in the general population. Furthermore, these previous studies have not explored the serum Cys C in relation to depressive symptoms and suicidal ideation.

In order to develop a better understanding of the potential association between serum Cys C levels and depressive symptoms, and to further explore its association with suicidal ideation, we assayed for Cys C in the general population.

## Materials and methods

### Participants

A total of 159 patients diagnosed with MDD were recruited from the Department of Psychiatry and Clinical Psychology at the People’s Hospital of Wuhan University from January 2019 to November 2020. Among them, 29 were excluded, 18 refused to participate in the examination, and 11 were unable to determine whether or not they had a family history of depression because their families had not been formally diagnosed. The remaining 130 patients were included in the final analysis. A total of 112 healthy volunteers were recruited healthy controls via the Internet in Hubei Province to form the control group. In total, the study included 130 patients with depression [aged 30.00 (20.75–50.00) years; 49 males] and 112 healthy controls without family history of depression or mental disorders [aged 31.00 (21.00–50.25) years; 44 males]. The diagnosis of depression was made according to the International Classification of Diseases-10 criteria (ICD-10) (https://icd.who.int/browse10/2016/en). Depressive symptoms were assessed with the 24-item Hamilton Depression Scale (HAMD-24) by a long-term trained psychologist [31]. Patients with a HAMD-24 test score ≥ 20 were included in this study. The Baker Suicidal Ideation Scale (SSI) was used to evaluate the presence of suicidal ideation [32, 33]. Exclusion criteria included: use of antidepressants, severe somatic and brain organic diseases, family history of psychiatric disorders and any other psychotic disorders, tumors, diabetes, kidney or heart diseases, acute inflammation and other causes of inflammation. The study protocol was approved by the Medical Ethics Review Committee of Renmin Hospital, Wuhan University, China (WDRY2021-K041). All participants signed a written informed consent before they were included in the study. All serum samples were obtained from the consenting volunteers. A smoker was defined as an individual who smoked either daily or occasionally in the 1 month preceding the time of survey [34]. Alcoholic consumption is often defined by whether or not drinking in the past year or six months or one month [35]. A traumatic life event is an extremely stressful event in which a person experiences or witnesses someone else’s of experience serious or even life-threatening injuries [36].

### Blood sampling and analysis

Participants fasted after 8:00 pm the day before the examination, and 5 ml of venous blood was collected from the elbow between 7:00 am to 9:00 am on the day before the examination. Serum specimens were obtained by centrifugation for 10 min at 3500 rpm and stored at − 80 °C until analysis. Serum Cys C levels were measured by Advia 2400 automatic biochemistry analyzer (Siemens, Erlangen, Germany; Serial Number: CA1258000740074). The Cys C reagent was purchased from Dako company in the United States (Cat. No.: LX004). Particle enhanced immunoturbidimetric assay was used for the detection of serum Cys C. Furthermore, serum levels of urea, creatinine (Cr), uric acid (UA), estimate glomerular filtration rate (eGFR), high-sensitivity C-reactive protein (hs-CRP) were also analyzed.

### Statistical analysis

All statistical analyses were performed using Graphpad Prism 7.0, IBM SPSS Statistics 22.0 and R version 3.6.3 (www.r-project.org). The means±standard deviation (SD) were used to describe the measurement data that obeyed the normal distribution, whereas, the median with the interquartile range (IQR) was used for data that did not obey the normal distribution. All categorical variables, such as gender, were expressed by frequencies (percent) and compared using the chi-squared test. Mann Whitney U test was used to compare the mean values between two the groups of non-normally distributed data. Independent sample t-test was used to compare the mean values between the two groups of normally distributed data. Generalized linear regression was performed to examine the relationship between serum levels of Cys C and HAMD-24 scores. Serum Cys C was divided into quartiles (quartile 4: ≥75th, quartile 2: 25–50th, quartile 3: 50–75th, quartile 1: < 25th percentile) [37]. Logistic regression was used to examine the associations between serum levels of Cys C and suicidal ideation, with quartile 1 serving as the reference category. The crude model had no adjustment. Model 1 was adjusted for age, gender distribution, smoking, alcohol consumption, family history of depression and traumatic life events. Model 2 was adjusted for the same variables as Model 1 as well as Urea, Cr, UA, eGFR and hs-CRP. To further investigate the relationship between serum Cys C levels and suicidal ideation, restricted cubic spline analysis was conducted in a fully adjusted model. A double-tailed *p* value < 0.05 was considered statistically significant.

## Results

The clinical characteristics of the MDD patients and the characteristics of the healthy controls are presented in Table 1. All the indicators assessed in this sample were included. The MDD and control groups did not significantly differ in terms of age [MDD: 30.00 (20.75–50.00) years; controls: 31.00 (21.00–50.25) years]. Furthermore, the MDD and control groups did not show any significant differences in terms of gender distribution, smoking, and alcohol consumption (all *p* > 0.05). The analysis indicated that serum Cys C levels were higher in MDD patients than in healthy controls (*p* = 0.001) (Table 1).

**Table 1:** Clinical characteristics of control group and MDD patients

Variable	MDD patients (*n* = 130)	Controls (*n* = 112)	Statistics	*P*
Age, year	30.00 (20.75–50.00)	31.00 (21.00–50.25)	*Z* = − 1.895	0.058
Gender, Male n (%)	49 (37.7%)	44 (39.3%)	χ2 = 0.065	0.799
Smoking n (%)	17 (13.1%)	12 (10.7%)	χ2 = 0.318	0.573
Alcohol consumption n (%)	7 (5.4%)	5 (4.5%)	χ2 = 0.108	0.742
Family history of depression n (%)	15 (11.5%)	–	–	–
Traumatic life events n (%)	12 (9.2%)	–	–	–
HAMD-24 score	24.00 (22.00–28.00)	–	–	–
SSI score^a^	8.00 (3.00–12.00)	–	–	–
Cys C (mg/L)	0.83 (0.75–0.93)	0.78 (0.73–0.85)	*Z* = − 3.314	0.001
Urea (mmol/L)	4.35 (3.57–5.29)	4.50 (3.73–5.38)	*Z* = − 0.849	0.396
Cr (μmol/L)	56.50 (50.00–67.00)	58.00 (47.25–70.00)	*Z* = − 0.171	0.865
UA (μmol/L)	306.50 (255.75–357.00)	301.00 (248.25–376.75)	*Z* = − 0.296	0.767
eGFR (mL/min/1.73m^2^)	110.84 (99.15–126.65)	115.76 (109.38–124.14)	*Z* = − 1.982	0.048
hs-CRP (mg/L)	0.14 (0.03–0.90)	0.11 (0.03–0.25)	*Z* = − 1.971	0.049

All data are presented as the interquartile range (IQR). The independent t-test and Mann Whitney U test were used for comparison of continuous data, and chi-squared test was used for proportions

^a^: the total SSI score; MDD: Major depressive disorder; HAMD-24: 24-item Hamilton Depression Scale; Cys C: serum Cystatin C; Cr: creatinine; UA: uric acid; hs-CRP: high-sensitivity C-reactive protein; eGFR: estimate glomerular filtration rate

Table 2 shows the relationship between serum Cys C level and HAMD-24 score. The crude regression coefficient of HAMD-24 score was 21.294 (95% confidence interval: 7.137, 35.451), indicating that serum Cys C level was positively correlated with HAMD-24 score (*p* = 0.003). After adjustment for age, gender distribution, smoking, alcohol consumption, family history of depression and traumatic life events in Model 1, higher serum Cys C levels were significantly related to a high HAMD-24 score (*p* = 0.012), with a regression coefficient of 20.497 (95%CI: 4.622, 36.371). This association remained statistically significant after additionally controlling for Urea, Cr, UA, eGFR and hs-CRP in Model 2 (*p* = 0.005). The fully adjusted regression coefficient was 30.339 (95%CI: 9.602, 51.077).

**Table 2:** Association of HAMD-24 scores with serum Cys C levels in MDD patients

Model	Serum Cys C (mg/L)		
	Standard Error	β (95%CI)	*P*-value
Crude	7.155	21.294 (7.137–35.451)	0.003
Model 1	8.109	20.497 (4.622–36.371)	0.012
Model 2	10.451	30.339 (9.602–51.077)	0.005

Generalized linear regression was performed to examine the relationship between serum levels of Cys C and HAMD-24 scores

Crude no adjustment

Model 1 adjusted for age (log-transformed), gender distribution, smoking, alcohol consumption, family history of depression and traumatic life events

Model 2 adjusted for the same variables as Model 1 as well as Urea (log-transformed), Cr (log-transformed), UA (log-transformed), eGFR (log-transformed) and hs-CRP (log-transformed)

In addition, the clinical manifestations of various depressive episodes in patients with MDD were analyzed. The results showed a non-significant statistical difference in the serum Cys C level between patients with or without the following clinical characteristics: family history of depression and previous depression (all *p* > 0.05). The serum Cys C concentration of female depressed patients was lower than that of males (*p* <  0.001). The concentration of serum Cys C in patients with suicidal ideation was higher than those without suicidal ideation (*p* = 0.001) (Table 3).

**Table 3:** Comparison of Cys C serum concentrations (mg/L) in MDD patients with different clinical picture of depressive episodes

MDD patients	Cys C (mg/L)	Statistics	*P*
Male (*n* = 49)	0.90 (0.81–0.98)	*Z* = −4.052	< 0.001
Female (*n* = 81)	0.80 (0.73–0.90)		
With family history of depression (*n* = 15)	0.85 ± 0.11	*t* = 0.019	0.985
Without family history of depression (*n* = 115)	0.85 ± 0.12		
With traumatic life events (*n* = 12)	0.94 (0.88–0.98)	*Z* = − 3.014	0.003
Without traumatic life events (*n* = 118)	0.82 (0.75–0.92)		
With first-episode depression (*n* = 78)	0.83 (0.76–0.93)	*Z* = − 0.525	0.599
With recurrent depression (*n* = 52)	0.84 (0.75–0.96)		
With suicidal ideation (*n* = 89)	0.86 ± 0.12	*t* = − 3.332	0.001
Without suicidal ideation (*n* = 41)	0.79 ± 0.11		

Data are expressed as mean ± SD or interquartile range (IQR); The independent t-test and Mann Whitney U test were used for comparison of continuous data, and chi-squared test was used for

proportions. MDD: Major depressive disorder; Cys C: serum Cystatin C

**Fig. 1:**
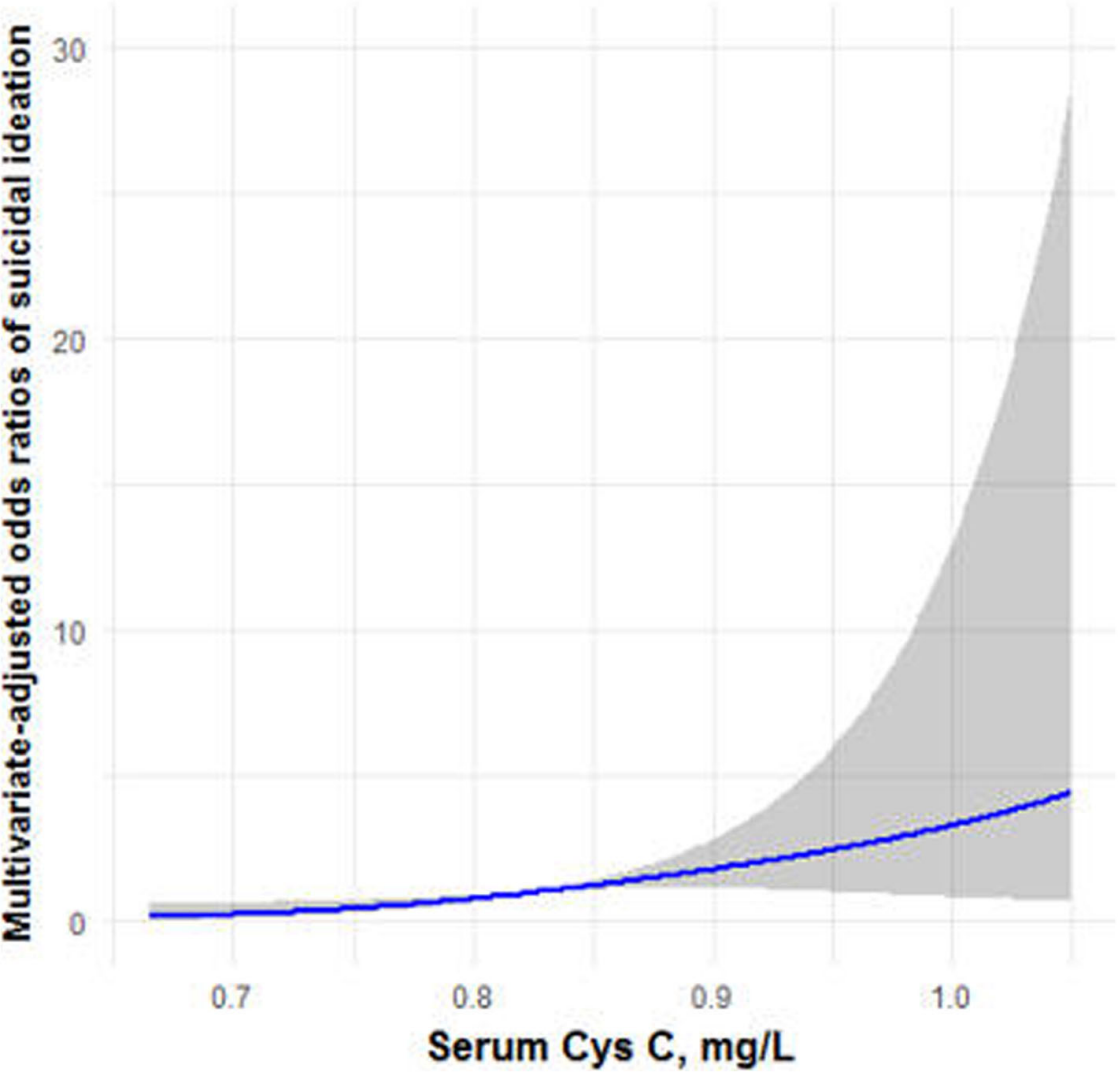
Restricted cubic spline model of the odds ratios of suicidal ideation with serum Cys C. The dashed lines represent the 95% confidence intervals. Cys C: serum Cystatin C. Serum Cys C level was positively correlated with suicidal ideation

In order to further analyze the correlation between suicidal ideation and serum Cys C level, we divided patients into Cys C quartiles and calculated odds ratios of suicidal ideation, taking patients in the first Cys C quartile as the reference (Table 4). The multivariate adjusted odds ratio for suicidal ideation showed an association between suicidal ideation and serum Cys C levels. Serum Cys C concentration was positively associated with suicidal ideation in the crude model (*p* = 0.011). The results were similar after adjustment for age, gender distribution, smoking, alcohol, family history of depression and traumatic life events in Model 1 (*p* = 0.011). Furthermore, this association remained statistically significant after additionally controlling for Urea, Cr, UA, eGFR and hs-CRP in Model 2 (*p* = 0.015). The fully adjusted OR of suicidal ideation in Model 2 was 9.753 (95%CI: 1.520, 62.575) in quartile 4 of serum Cys C levels (the highest) versus quartile 1 (the lowest). At the same time, restrictive cubic spline analysis also showed that serum Cys C level was positively correlated with suicidal ideation (Fig. 1).

**Table 4:** Association of suicidal ideation with serum Cys C in MDD patients

Cys C quartile	n	Conc range, mg/L	EXP(β), OR(95%CI)		
			Crude	Model 1	Model 2
Quartile 1 (low)	36	< −0.12	Reference	Reference	Reference
Quartile 2	29	−0.12 to − 0.08	0.780, 2.182 (0.794-5.994)	1.102, 3.009 (0.988-9.162)	1.205, 3.335 (0.949-11.722)
Quartile 3	35	−0.08 to − 0.03	0.965, 2.625 (0.888-7.762)	1.202, 3.325 (0.998-11.082)	1.280, 3.595 (0.874-14.779)
Quartile 4 (high)	30	> −0.03	1.421, 4.143 (1.389-12.352)	1.733, 5.657 (1.531-20.902)	2.278, 9.753 (1.520-62.575)
*P* for trend			0.011	0.011	0.015

Logistic regression was used to examine the associations between serum levels of Cys C and suicidal ideation. Serum Cys C was divided into quartiles (quartile 4: ≥75th, quartile 3: 50–75th, quartile 2: 25–50th, quartile 1: < 25th percentile)

Crude no adjustment

Model 1 adjusted for age (log-transformed), gender distribution, smoking, alcohol consumption, family history of depression and traumatic life events

Model 2 adjusted for the same variables as Model 1 as well as Urea (log-transformed), Cr (log-transformed), UA (log-transformed), eGFR (log-transformed) and hs-CRP (log-transformed)

## Discussion

MDD, with severe impairment in cognitive and social functioning, is a common and complex psychiatric disorder characterized by persistent low mood, loss of interest and lack of energy. MDD has been considered as a multifactorial disorder with neurological, genetic and environmental factors contributing to the overall risk [38–40]. However, the mechanisms of these risk factors are still unknown.

In this retrospective study of MDD patients, we found a positive correlation between serum Cys C level and HAMD-24 score, which remained significant after controlling for potential confounding factors. These effects were independent of gender distribution, age, smoking, alcohol consumption, family history of depression, traumatic life events, Urea, Cr, UA, eGFR and hs-CRP. Similar results were observed between serum Cys C levels and suicidal ideation when adjusting for all of the above covariates. To the best of our knowledge, this is the first study that investigates the associations between serum Cys C levels and depressive symptoms and suicidal ideation.

Several studies have examined a potential link between Cys C levels and depression [29, 30, 41]. A study that enrolled 1440 Chinese older adults (> 60 years old) found a detrimental relationship between high serum Cys C levels and the risk of depression [29]. Another prospective cohort study of 11,847 Chinese people (> 45 years old) by Li H et al. [41] showed that high levels of Cys C were associated with an increased risk of depression. They calculated the risk of depression using a corrected Poission regression model and found that the association between serum Cys C and the risk of depression remained significant after adjustment for multiple covariance. Consistent with the above findings, our results show that serum Cys C not only is associated with depression; but also demonstrated - for the first time, that high serum Cys C is closely related to the severity of depressive symptoms. This latter association persists after controlling for multiple potentially confounding variables. We speculate that cystatin C may play an important role in the pathogenesis of depression.

Cys C exerts its biological functions in multiple aspects of biological activity and nerve physiology [42] and therefore, it may affect the risk of depression in a variety of ways. First, serum Cys C is associated with inflammation [43] and affects neutrophils migration [44, 45]. This promotes the secretion of pro-inflammatory cytokines such as IFN-γ and TNF [46], which may impair the function of the brain serotonin system, then activate the hypothalamic-pituitary-adrenal axis, and eventually cause depressive symptoms through the inflammatory pathway. Second, another mechanism by which Cys C may influence the risk of depression is through apoptosis. This can directly or indirectly lead to depression [26]. Cys C induces neuronal apoptosis by increasing the level of active caspinase-9 protein and decreasing the level of B-cell leukemia 2 (Bcl-2) in the Jun-terminal kinase (JNK) dependent pathway [47], which may be an important risk factor for depression [48]. The last possible mechanism is that cystatin C is related to oxidative stress, which plays an important role in depression [49]. Studies have previously shown that oxidative stress can up-regulate the concentration of Cys C in the nervous and cardiovascular system. The increase of Cys C level induced by oxidative stress may be mediated by reactive oxygen species (including hydrogen peroxide, superoxide anion, hydroxyl radical, etc.), but the specific mechanism is still unclear [50, 51]. The abnormally elevated level of Cys C in MDD patients may be explained by the above mechanisms.

Interestingly, the present study also found that the serum Cys C level of patients with suicidal ideation was higher than that of patients without suicidal ideation, suggesting that serum Cys C level has a significant relationship with suicidal ideation in MDD patients. Although, a wealth of previous studies has provided valuable insights on the association between serum Cys C and depression; no one has studied the relationship between Cys C and suicidal ideation in patients with depression. The present study found that high serum Cys C level is a risk factor for suicidal ideation, suggesting that Cys C may be involved in the pathogenesis of suicidal ideation in patients with depression, but the specific mechanism has not yet been clarified.

One potential pathway could be that changes in Cys C levels may related to increased neuronal inflammation, which may increase suicidal tendencies in people with depression. Some inflammatory factors, such as IL-6 and TNF-α, affect the risk of suicide in patients with depression by impacting the serotonergic system [52–54]. A second potential pathway, could be that Cys C plays an important role in inducing neuronal apoptosis [15, 47]; and polyamine-mediated apoptosis leads to the reduction of neurodegenerative gray matter volume, which is related to the susceptibility to suicide [48]. A third mechanism by which Cys C may influence suicidal ideation in MDD patients is through destruction of white matter. Mutations in the CST3 gene, which encodes cystatin C, increase cathepsin activity, leading to white matter damage [55], which may be related to suicide [56]. A fourth mechanism may be through brain amyloid deposition: Cys C, as one of the few amyloid-forming proteins, can also be deposited along with β-amyloid in the microscopic blood vessel walls of the brain, increasing cerebrovascular damage. These amyloid deposits have been linked to treatment-resistant depression and may play a significant role in the risk of suicide in depression [57]. In the present study, the relationship between changes of Cys C level and suicidal ideation in MDD patients may be explained by the above mechanisms, however, the specific mechanism remains to be clarified in future studies.

Although our results add useful knowledge to the field, it is important to note some limitations, such as: the sample size was relatively small, and there were many interfering factors in the results. For example, the relationship between Cys C level and suicidal ideation was analyzed without considering other confounding factors. Other limitations include: the relatively limited source of the study, which involved only some regions; the fact that prescribed medications were not controlled; the different eligibility criteria applied to study patients and controls may have introduced systematic biases. In addition, it was a case-control study and therefore, cross-sectionality is a major limitation, which meant that the study could only show associations and not causality.

Despite these limitations, our results clearly demonstrate that serum Cys C levels are associated with the depressive symptoms and suicidal ideation in MDD patients. This may be a potential biomarker for depression and suicidal ideation. However, the current research on the mechanism of Cys C and depression and suicidal ideation is not comprehensive. Therefore, larger sample size studies will still be needed to demonstrate a more robust relationship between serum Cys C and depression and suicidal ideation.

## Data Availability

The datasets used and/or analysed during the current study are available from the corresponding author on reasonable request.

## Abbreviations

Cys C: Serum Cystatin C
MDD: major depressive disorder
HAMD-24: 24-item Hamilton Depression Scale
WHO: World Health Organization
ICD-10: International Classification of Diseases-10 criteria
SI: suicidal ideation
Cr: creatinine
UA: uric acid
hs-CRP: high-sensitivity C-reactive protein
eGFR: estimate glomerular filtration rate
